# CFD-DEM model of plugging in flow with cohesive particles

**DOI:** 10.1038/s41598-023-44202-7

**Published:** 2023-10-11

**Authors:** Nazerke Saparbayeva, Boris V. Balakin

**Affiliations:** Department of Mechanical and Marine Engineering, Western Norway University of Applied Sciences, 5063 Bergen, Norway

**Keywords:** Fluid dynamics, Computational science

## Abstract

Plugging in flows with cohesive particles is crucial in many industrial and real-life applications such as hemodynamics, water distribution, and petroleum flow assurance. Although probabilistic models for plugging risk estimation are presented in the literature, multiple details of the process remain unclear. In this paper, we present a CFD-DEM model of plugging validated against several experimental benchmarks. Using the simulations, we consider the process of plugging in a slurry of ice in decane, focusing on inter-particle collisions and plugging dynamics. We conduct a parametric study altering the Reynolds number (3000...9000), particle concentration (1.6...7.3%), and surface energy (21...541 mJ/m$^2$). We note the process possesses complex non-linear behaviour for the cases where particle-wall adhesion reduces by more than 20% relative to inter-particle cohesion. Finally, we demonstrate how the simulation results match the flow maps based on the third-party experiments.

## Introduction

Cohesive particles can significantly impact the morphology of multiphase flows. Sticking to each other and walls, the particles build an obstruction or plug in closed channels. The plugging is crucial in many applied fluid mechanics problems: flows in porous media, hemodynamics, and suspension rheology^1^. The industrial relevance of the problems concerns petroleum, pharmaceutical, chemical, and food industries. More globally, the process of plugging is relevant for behaviour models of animals^1^. The plugging is dependent on the flow field, the number of particles, their cohesivity, and contact behaviour. However, due to the complexity of inter-particle and particle-fluid interactions, no reliable methodology is used to predict the plug formation process. Experimental flow maps enable evaluation of plugging risks for a limited interval of flow conditions^2^. Therefore, a better theoretical understanding of the fluid mechanics of plugging is required to extend and update the existing empirical correlations.

For this reason, numerical models of plugging based on the principles of computational fluid dynamics (CFD) are developed. About a decade ago, simplified models of plugging were developed for petroleum^3,4^ and medical applications^5^. Eskin et al.^3^ considered the process of asphaltene deposition in petroleum pipes using the advection-diffusion method coupled with the population balance approach, which simulated the agglomeration of asphaltene particles. Cohesive interactions of particles were modelled using empirical correlations where coefficients were fitted to a smaller-scale experiment. The model could simulate a uniform, continuous deposit blocking $\sim 30\%$ of the pipe-cross section with no overall flow reduction. A similar approach was used by Rukhlenko et al.^5^ to simulate thrombosis in a blood vessel. This work used a single-phase CFD model coupled with a population balance approach to define a porous zone where the blood coagulation happened and resulting fibrin structures were deposited. The simulations resulted in flow maps highlighting intervals of vessel sizes and Reynolds numbers where the thrombus formation was most probable. Labois et al.^4^ presented a more complex three-phase Eulerian-Eulerian model of gas hydrate deposition in the subsea conditions of gas leakage. An innovative aspect of the proposed simulation approach was defining the second, stationary hydrate phase generated from the moving hydrate phase when it adhered to a structure. However, this transition’s details were unclear as the authors did not present sufficient details of the simulation approach. The considered models^3,4^ were based on empirical closure relations and required input of several fitting parameters determined experimentally. Moreover, none of the models was verified against a relevant experimental benchmark.

A more accurate simulation approach would consider interactions of individual particles during the plugging. In this case, an Eulerian CFD model is combined with a Lagrangian Discrete Element Method (DEM) capable of reproducing inter-particle collisions. Several works used this method to study the clogging of relatively large particles at local flow restrictions. They considered how various parameters affect plugging, such as particle size, concentration, velocity, and shape. The study by Hilton et al.^6^ focused on the effect of particle shape on the volumetric dynamics of pneumatic transport systems. This model treated collisions of $\sim 400$-$\upmu \text {m}$ particles using a standard soft-sphere model with the so-termed *spring, dash-pot, and slider*. The model, validated against experiments, was capable of depicting particle slugs blocking the entire cross-section of the pipe. Interestingly, the simulations demonstrated that spherical particles or those close to spherical shape led to stable flow at volume fractions around 60%, while ellipsoidal particles led to slug formation when the ellipticity was under 0.7 or above 1.3. Yang et al. used the CFD-DEM method to examine the plugging of particles in the shale pores^7^. Their findings showed that particle size and concentration are crucial in plugging efficiency. Additionally, the authors noted that the particle velocity, roughness, and tortuosity significantly affect the blockage of the pores. Ma et al.^8^ utilized the CFD-DEM approach to investigate the blocking mechanism in pre-packed gravel screens commonly used in oil and gas wells. The study found that the size and concentration of large particles affected blockage, and increasing the screen’s porosity could reduce particle accumulation. Mondal et al.^9^ studied the behaviour of particulate suspensions at a constriction for concentrations $<50\%$ using the CFD-DEM method. Their research revealed that the resolved approach is suitable for systems where the particle size is comparable to the flow geometry. They also investigated the phenomenon of multi-particle hydrodynamic bridging. They found that the probability of clogging increases with particle volume concentration, suggesting a critical particle volume concentration for the spontaneous formation of bridges. The critical concentration was in the interval of 7–32% and dependent on the ratio between the particle size and the diameter of the flow restriction. A similar system was considered by Xu et al.^10^, who modelled the clogging of a constriction by polydisperse sand particles. They examined how particle size and shape affect clogging probability and found that the largest particles from the size distribution formed a particle jamming arch. The probability of clogging approached unity when $d_{84}/d_{16}>1.8$. None of the abovementioned CFD-DEM studies considered plugging by cohesive particles.

Several studies investigated the process of plugging with cohesive particles. Shao et al.^11^ analysed the mechanism of clogging in microchannels by the gas-solid flow where the particles were sticky due to liquid bridges and van-der-Waals interactions. The size of the channel was comparable with the size of the particle ($\sim \text {d}{_p}$), and the particles’ Young modulus was lower than the respective parameter of most solid materials. The plugging event was defined as the channel blockage by a particle with no simultaneous stagnation in the gas phase. The simulation resulted in flow maps illustrating how the plugging depends on the particle Stokes number (St) and the Weber (We) number of the bridge. As follows from the maps, plugging took place for $\text {St}<3.5$ and $\text {We}<10$. Duan et al.^12^ simulated an industry-relevant problem of methane hydrate blockage at two different flow restrictions in a water-dominant system. The CFD-DEM model treated cohesive collisions between particles, combining the Hertz-Mindlin approach with Johnson–Kendall–Roberts (JKR) cohesion model. A simplified validation of the model was performed comparing with the experimental pressure gradients for cases with no plugs in a homogeneous flow regime. The model reproduced the formation of a sand watch-like deposit at the restriction with no total flow stop by this deposition. The deposit size was proportional to the flow velocity and the size of solid particles. Wang et al.^13^ used a CFD-DEM approach to model gas-solid flow with hydrate particles through a pipe with varying diameters. The model simulated cohesive interactions using the JKR approach. Although the Young modulus of the particles was significantly below referent values for gas hydrates, and the surface energy of the particle is not provided in the paper, the simulation results were compared surprisingly well with the experiments. Further, the authors considered how the particles’ deposition efficiency depends on mean flow velocity and the particle-to-diameter size ratio. The deposition efficiency appeared in the interval 2% to 34%, meaning that the model did not reproduce the process of plugging.

Concluding the introductory part, we note very few models are tailored to accurately predict mechanisms that govern the plug formation process. The models strongly rely on empiricism or fail to reproduce the process as it happens for most real-life situations: a full flow stop caused by a sticky deposit of particles. The models are not validated against a plugging experiment. This study addresses the challenges by introducing a CFD-DEM model validated against a well-defined experimental benchmark by Struchalin et al.^2^ for plugging in an ice-decane slurry. Previously, in our study, Saparbayeva et al.^14^, we utilized the CFD-DEM model to investigate the ice-ice cohesive collision and obtained insights helpful to develop the CFD-DEM model for the entire flow. For the first time, the model reproduces the plug formation process in sufficient detail and demonstrates how the plugging depends on the critical parameters of the process.

## Methods

### Model description

The CFD-DEM approach employed an Eulerian-Lagrangian framework to solve the fluid and solid phases independently. The fluid phase was described by a system of turbulent, incompressible Navier-Stokes equations^15,16^:

1 $$\begin{aligned} D \phi / D t = 0; \;\; D\left( \rho \phi \vec {u}\right) / D t = - \phi \nabla p + \phi (\mu +\mu ^{t}) \nabla ^2 \vec {u} + \phi \rho \vec {g}- \vec {F}_{p}, \end{aligned}$$

where $\phi$ is the volume fraction of the continuous phase, $\vec {u}$ is the velocity of the continuous phase, $\rho$ is the density of the continuous phase, *p* is the pressure, $\mu$ and $\mu ^{t}$ are molecular and turbulent viscosity, $\vec {g}$ is the acceleration due to gravity. The standard k-epsilon turbulence model computes the turbulent viscosity^16^. We further assumed that the heat transfer with the ambient environment did not sufficiently influence the properties of the continuous phase and then excluded the energy equation from the analysis. The combined effect of the drag and lift forces exerted by DEM particles in the continuous phase is presented by $\vec {F}_{p}$^14^ for a computational cell.

Newton’s second law governs the motion of the $\text {i}$th DEM particle^15^:

2 $$\begin{aligned} m_i \frac{{\textrm{d}}\vec{v_i}}{\textrm{d} t} = \vec {f}_{p,i} + \vec {f}_{l,i} + m_i \vec {g} - (m_i/\rho _p) \nabla p + \sum _{j=1,N} \vec {f}_{i,j}, \end{aligned}$$

where $m_i$ is the mass of the particle, $\vec {v_i}$ is the particle’s velocity, $\rho _p$ is the density of the particle. The drag force is determined as $\vec {f}_{p,i} = \frac{\pi }{2} r^2_{i} c_{D,i} \rho (\vec {u} - \vec {v_i}) |\vec {u} - \vec {v_i}|$,where $r_i=200$ $\upmu \text {m}$ is the radius of the particle, $c_{D,i}$ is Schiller–Naumann’s drag coefficient^14,17^. The lift force is calculated as $\vec {f}_{l,i}=c_{l}\rho \pi r_i^3 \left( \vec {u}-\vec {v_i}\right) \times \vec {\omega }$ with lift coefficient $c_{l}$ calculated according to Sommerfeld’s expression^18^ and curl of the fluid velocity $\vec {\omega }=\bigtriangledown \times \vec {u}$. The contact forces induced due to the collision with the *N* of $\text {j}$th neighbour particles (or the walls) are given by $\vec {f}_{i,j}$. The DEM solver activates this term when the particles are expected to contact their collision pairs at the next temporal substep of the DEM model. The particle rotation is calculated by accounting for the described forces. This is in detail described in Saparbayeva et al.^14^

The Hertz-Mindlin contact model accounting for cohesion determines the contact forces acting between particles and walls during the collision. They are given in normal (*n*) and tangential (*t*) directions relative to the plane of collision whose normal points from the $\text {i}$th particle^14,19^:

3 $$\begin{aligned} \vec {f}_{i,j}=F^{(n)}_{i,j}\vec {n}+F^{(t)}_{ij}\vec {t} \end{aligned}$$

The contact force in the normal direction can be expressed as^14^:

4 $$\begin{aligned} F^{(n)}_{i,j} = -K^{(n)} \delta ^{(n)} - N^{(n)} v^{(n)}_{r,i} +F_{C} \end{aligned}$$

where $\delta ^{(n)}$ is the particle-to-wall overlap distance, $v_{r,i}$ is the inter-particle relative velocity, *K* and *N* represent the stiffness and the damping coefficients. These parameters depend on the particles’ mechanical properties: the Young modulus, Poisson’s ratio, and the coefficient of restitution (COR). The cohesive force is computed using the JKR approach^20^ $F_{c}= 1.5 \pi r_i \gamma _i$, where $\gamma$ is the work of cohesion ($\gamma _{ice}$) or the work of the adhesion to walls ($\gamma _{wall}$). The tangential component of the contact force is determined in a similar to Eq. (4) fashion yet excluding the cohesive interaction $F^{(t)}_{i,j} = -K^{(t)} \delta ^{(t)} - N^{(t)} v^{(t)}_{r,i}$. A detailed description of the contact treatment is presented in Saparbayeva et al.^14^

**Figure 1:**
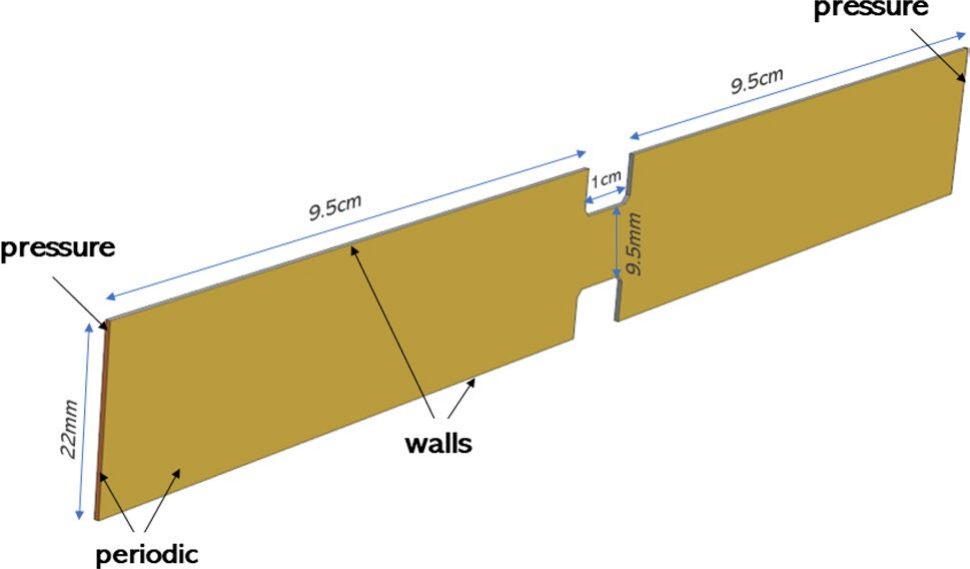
Geometry and boundaries.

### Boundaries and mesh

The numerical model was developed in the commercial CFD-package STAR-CCM+ 2210 (specifically version 17.06.007). To tailor the simulation to the specific needs of our research, we extended its capabilities by incorporating user-written codes, known as ’field functions’. These field functions played a crucial role in our work by allowing us to modify the default settings and configurations of the standard model. The geometry of the computational domain resembles the test section of the experimental flow loop described in Struchalin et al.^2^: 20 cm long pipe with an internal diameter of 22 mm contains a 1 cm long orifice blocking 80% of the pipe cross-section. In the experiments, the orifice was used to induce plugging in the test section. To conserve computational costs, we produced two geometries: a full-scale 3D test section, which was further sliced to a quasi-2D element bounded by periodic boundaries in a horizontal direction orthogonal to the main flow. The thickness of the slice was equal to 3 diameters of the particle. The periodic boundaries recycled the secondary flow and particles in this direction. As presented in Fig. 1, other boundaries include the pressure at the inlet and outlet of the pipe, and the rest of the surfaces are no-slip walls. The computational mesh was made of 8 $\text {mm}^3$ rectangular volumes. A rather coarse mesh resulted in elevated $\text {Y}+<6$. This meshing was chosen to ensure the Lagrangian particles were subgrid^17^, which also complies with the software developers’ guidelines^16^. For the current flow geometry, we did a mesh independence study. The analysis was conducted for mesh sizes ranging from 1 to 3 mm with a step size of 0.5 mm. When changing the mesh size, we noted a low statistical spread of plugging dynamics at $\sim 15\%$. Finally, we tested how a fully 3D case differs from a quasi-2D simplified model. To speed up the formation of the plug, we set the cohesion to the maximum 95% experimental value^21^ and used $\gamma _{ice}=\gamma _{wall}=541$ $\text {mJ}/\text {m}^2$. The difference was evaluated in terms of the rate of plug formation. The simulations revealed that the 2D results compared well to the 3D simulation with an average discrepancy of about 8%. However, we note the formation of more massive particle slugs in the 3D model. The 2D model used 142 times less computational time.

### Model settings

Multiple parameters of the model were set according to experimental conditions to reproduce plugging experiments. The process of plugging took place for $\sim 100$ s, and the temperature in the cross-section linearly increased from $-1.1$ to $-0.6^{\circ }\text {C}$ during the process. The heating of the flow was due to the particle-wall friction. The molecular properties of the phases were set for the time-average temperature according to the NIST database^22^. Since plugging resulted in a continuous reduction of flow rate, a flow regime transition was expected at the end of the process. The model simulated this effect by scaling the turbulent viscosity to zero when the flow Reynolds number fell below 2300. Table 1 presents the parameters of the model.

**Table 1:** Model parameters.

Diameter of particles $d_i$	400 $\upmu$m
Density of fluid $\rho$	747 kg/$\text {m}^3$
Density of particles $\rho _p$	916 kg/$\text {m}^3$
Fluid viscosity $\mu$	1.25 mPa s
Normal COR $\varepsilon _n$^23^	$0.8+1.8\ln x_{c}/x_{0}/\text {St}_{0}$, 0.63${}^{\textrm{a}}$, 0.8${}^{\textrm{b}}$
Tangential COR $\varepsilon _t$	0.8
Sliding friction coefficient *f*	$-0.015T + 0.574$, 0.1–0.6$^b$
Rolling friction coefficient $\mu _r$	0.001
Surface energy $\gamma$	$0.280+0.061T$, 0.541$^b$ J/$\text {m}^2$
Young’s modulus $E_p$	0.1 MPa
Poisson’s ratio $\nu _p$	0.36

Relative Stokes number $\text {St}_{0} = m_i v_{r,i}/6 \pi \mu r_i^2$^23^, $x/x_0$=10$^{-3}$, *T* is the experimental temperature in $^0$C. ${}^{\textrm{a}}$Asymptotic value, ${}^{\textrm{b}}$Simplified simulations.

For the mechanical properties of ice, we used the approach described in detail by Saparbayeva et al.^14^. We computed the normal coefficient of restitution $\varepsilon _n$ accounting for lubrication force from decane using the method proposed by Joseph et al.^23^ for particle Stokes numbers beyond 17. For the lower Stokes numbers, $\varepsilon _n$ was set at 0.05. The tangential coefficient of restitution was assumed to be unaffected by lubrication and then equal to the so-termed “dry” value $\varepsilon _t=0.8$^24^. Poisson’s ratio for ice was set as 0.36^25^. To limit the computational costs by increasing the DEM temporal sub-stepping^14,16^, the Young modulus of the particle was artificially reduced below the real values^26^ to 0.1 MPa. We carried out simulations to test the influence of this parameter on the dynamics of the process. The model was weakly sensitive to the increase of the Young modulus by 3 orders as the average change pipe blockage dynamics was $\sim 30\%$ while resulting in a 15-fold increase of the computational time. Although the rolling resistance may be significant in contact interactions^27^, the rolling friction coefficient was set at a low value of 0.001. Additional experiments are required to determine this parameter accurately for interactions of ice in decane. The default mechanical properties of glass^16^ were set for the walls of the computational domain.

We extended the model to more effectively account for temperature-dependent variables, including friction and cohesion, which previous studies have identified as significant factors influencing ice collisions^14^. The experimental temperature log was imported into the model. The cohesive surface energy was set linearly increasing with the temperature. For this, we interpolated experimental measurements for ice in decane presented by Yang et al.^21^. In the interpolation, we used data points obtained in the interval $-4.0\ldots -1.5 ^{\circ }\text {C}$ where $\gamma _{ice}\sim 21\ldots 172\,\text {mJ}/\text {m}^2$. The cohesive energy is calculated in the JKR limit from the micromechanical force measurements reported by Yang et al.^21^. We note that these values of cohesion energy are about 3-orders greater than the cohesion of clotted blood particles^28^.

A similar interpolation was conducted for the coefficient of friction *fr* based on the data from Sukhorukov^25^. For the interpolation, we used measurements taken in the interval $-8.0\ldots 1.8 ^{\circ }\text {C}$ for the shortest contact time between ice surfaces. The friction coefficient was in the interval $\sim 0.60\ldots 0.69$. The friction coefficient reduced with the temperature.

The adhesive energy of ice to the walls of the test section filled with decane and the coefficient of friction with the walls are not explicitly available in the literature. According to Aspenes et al.^29^, the adhesion is proportional to the free energy of the solid surface. The free energy of the walls^29^ is lower than the cohesive energy of ice^21^. The friction coefficient of ice at the pipe material is also lower than the ice-to-ice friction^25^. Therefore, we explored how the ratio of the ice-wall adhesion to the ice-ice cohesion $c_r=\gamma _{ice}/\gamma _{wall}<1$ influences the simulation results. We also noted that ice adhesion to different materials reduces with temperature^30,31^. Therefore, as a conservative estimate, the adhesion was set as a constant proportional to the initial value of cohesion. We also assumed that the friction coefficients between the particles and the walls were scaled proportionally to $c_r$.

The pressure at the inlet was specified to reproduce the experimental mass flow with neutrally buoyant and non-cohesive particles, which was determined in separate calibration simulations. Zero velocity and pressure fields were used as initial conditions for simulations. Initially, the flow field in the test section was established for about 2 s to the experimental value without DEM particles. In this way, we prevented the formation of particulate deposits during the start-up phase of the process. Then, the particles were injected at the inlet at 13 000 1/s, corresponding to the experimental volume fraction. To avoid phase slip at the inlet, mean flow velocity was continuously monitored and over-prescribed as the inlet velocity of DEM particles. In this model, we assumed that the significant deposition of the particles took place in the test section and neglected possible deposition in the rest of the experimental system. Moreover, due to the large volume of the system compared with the volume of the test section, we assumed that the deposition of the particles in the test section did not significantly influence the volume fraction of the particle at the inlet.

We used the SIMPLE in STAR-CCM+^14,32^ to solve governing equations for the continuous phase. The following relaxation coefficients were set for the solver: 0.8 velocity, 0.2 pressure, 0.9 volume fraction, and 0.9 turbulence model. The equations were spatially discretized using upwind discretization in space and the implicit Euler method (2nd order) in time. The time step was 0.5 ms. The time step of the DEM model parameter was set at 20% of the duration of the Rayleigh wave propagation through the particle^16,33^. The absolute values of the DEM time step were $\sim 20 \upmu \text {s}$.

## Results and discussion

### Model validation

It is noteworthy to highlight the significance of previous studies as they have contributed to our understanding of particle deposition and cohesive interactions in a pipe flow. We validated the applied CFD-DEM model with no account for cohesion in Eq. (4). First, we reproduced the blockage for the process described in Mondal et al.^9^. The obtained results reproduced clogging of the flow channel at a particle volume concentration of $13\%$  close to the value of $10\%$ reported in the original work. In addition, by applying the non-cohesive CFD-DEM approach to experimental data sets, we successfully simulated dune formation in a microchannel during horizontal hydraulic transport^34^. The predicted velocity of the dunes exhibited a $10.7\%$ deviation from the corresponding experimental values. Cohesive particles significantly contribute to block formation in this study. It is worth noting a related study using our CFD-DEM model with included cohesion accurately predicted restitution coefficients, with a $10\%$ average deviation from experimental data^14^.

**Figure 2:**
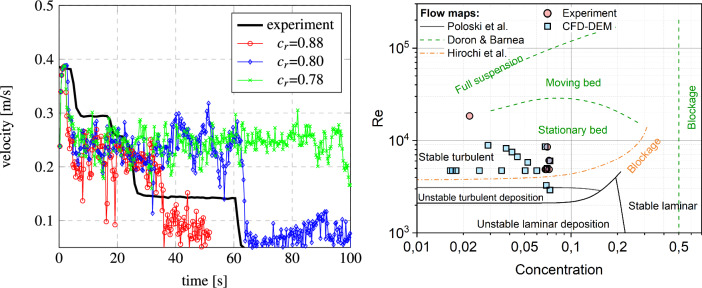
CFD-DEM model compared with experimental results: (left) average flow velocity as a function of time for difference cohesion to adhesion ratios $c_r=\gamma _{wall}/\gamma _{ice}$ compared to Struchalin et al.^2^ at Re = 4996, $\phi _p=6.8\%$; (right) flow maps by Poloski et al.^35^, Doron and Barnea^36^, Hirochi et al.^37^, Struchalin et al.^2^ (Experiment) compared with the CFD-DEM predictions at $c_r=1$, $\varepsilon _n=0.63$, $fr=0.6$.

**Figure 3:**
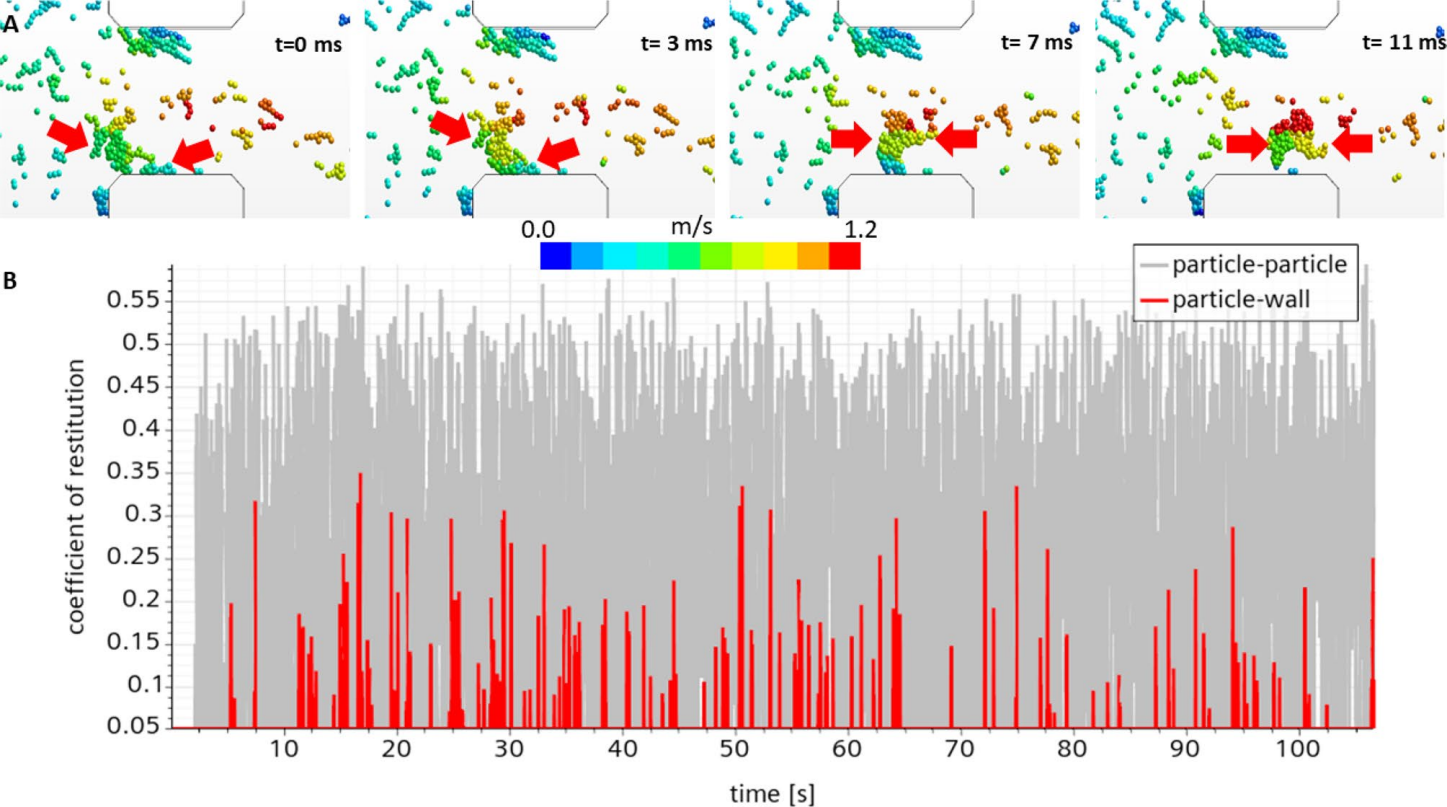
(**A**) Scrubbing of deposited particles from the walls. Color scale denotes the magnitude of the continuous phase velocity. (**B**) Coefficient of restitution for particle-particle and particle-wall collisions.

The CFD-DEM model was then validated against the experiment by Struchalin et al.^2^. The model reproduced experimental case 2 from Struchalin et al.^2^, where a plug was formed in the test section at an initial flow rate of 400 kg/h and a particle concentration of 6.8%. For this system, cohesion was known from the experiments by Yang et al.^21^ while the adhesion and the friction of the particles at the walls were fitted parameters. They were tuned proportionally to the cohesion to match the experiment. The left plot in Fig. 2 demonstrates how the mean flow velocity changed with time during the plugging of the pipe. We note a slug-like behaviour of the flow in the CFD-DEM model for low adhesion cases. This is connected to more sticky particles re-dispersed deposits formed at less sticky walls. This phenomenon is illustrated in Fig. 3A. As presented in Fig. 3B, in these simulations, the maximum coefficients of restitution for the particle-particle and particle-wall conditions reduced from 0.8 to 0.55 and 0.35 due to the lubrication effects (see Methods). The reduced restitution coefficients contribute to the faster plugging of the test section.

The best correspondence to the experiments was obtained when adhesion and friction were $\sim 88\%$ of the cohesion; the average discrepancy of the model was 25%. For lower adhesion, the simulated velocity sharply reduced to $\sim 50\%$ of the initial values; the flow rate remained at this value for 60-100 s without a significant reduction due to the re-dispersion of the deposited particles. However, as the model incorporated the experimental temperature log and the cohesion was set dependent on the temperature, this parameter increased with time. Large particle slugs formed when the cohesion significantly increased. At the end of the process, the slugs blocked the orifice. For the high adhesion values, the step-wise shape of the experimental curve corresponded to the experimental. The differences are addressed in the secondary deposition and jamming in other parts of the experimental system that are not modelled in CFD-DEM (e.g. pump, mixing tank, flow meter). The respective growth of the secondary flow resistance in non-modelled locations contributes to the rate of flow velocity reduction.

It is interesting to consider how the CFD-DEM model reproduced the third-party flow maps. We present this information in Fig. 2 (right). Here, we collect data for flow regimes in horizontal flows of slurries and suspensions of particles. To exclude the influence of pipe material, we set $c_r=1$. Next, speeding up the simulations, $fr=0.6$, which is the maximum for the considered system, and $\varepsilon _n=0.63$ corresponding to the maximum relative velocity. The experiments are compared with cases where simulations resulted in plugging of the test section. As in the experiments by Strcuhalin et al.^2^, the model reveals plugging in flow regimes where stationary deposits with no plugging are supposed to be formed. This is an expected trend since the maps are developed for particles with significantly lower cohesion and adhesion. However, the model corresponds to the referent experiment demonstrating plugs and the flow rates below the experimental and comparable concentrations.

### Plugging dynamics

**Figure 4:**
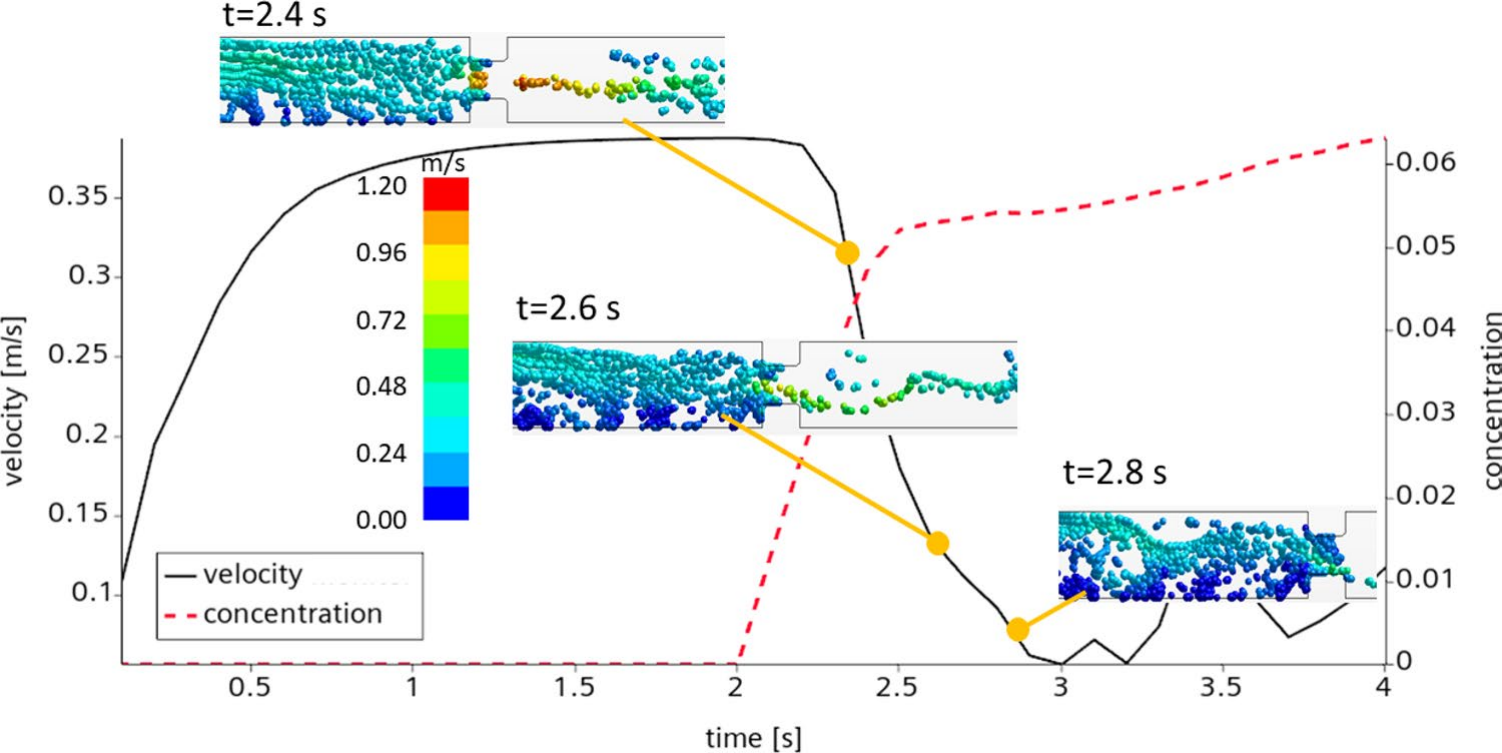
Dynamics of plug formation. Surface energy $\gamma _{ice}=\gamma _{wall}=541\,\text {J}/\text {m}^2$, mean flow velocity ${\overline{u}}=0.39$ m/s, particle concentration $\phi _p=5.9\%$, coefficient of restitution $\varepsilon _n=0.8$, $fr=0.1$. Flow direction from left to right, color scale denotes the magnitude of the continuous phase velocity.

Figure 4 considers the dynamics of blockage in more detail. Here, to shorten the simulation and limit the re-dispersion of the particles, we set the cohesion to the maximum experimental value $541\,\text {mJ}/\text {m}^2$ according to Yang et al.^21^, neglect lubrication, and minimize the friction. The simulation results reveal a continuous reduction of mean flow velocity after the particles were injected at t = 2 s. Shortly after the injection, the particles were driven to the bottom of the pipe, where they adhered to the walls and formed stationary deposits. The volume fraction of the particles in the deposits was about 50%, close to the packing limit reported in the benchmark study^2^. Once the deposition progressed, the particles experienced inertial deposition at the surface of the orifice for $\text {t}=2.6$ s. Smaller deposits were built both at the frontal part of the orifice and directly in its throttle. The deposition resulted in an elevated flow resistance, which led to a dramatic flow reduction for a fixed pumping pressure drop. As in the experiments^2^, the plug was formed at the very end of the process due to trapping the bottom deposits with those formed at the orifice. After the stationary plug was formed, the flow experienced low-magnitude oscillations. This chocking happens due to the inertial motion of particles in the upflow part of the test section.

### Sensitivity analysis

**Figure 5:**
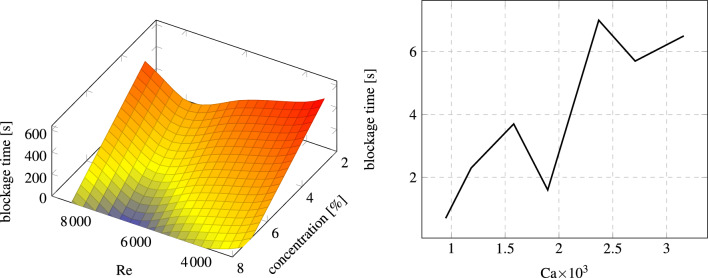
Blockage time as a function of flow Reynolds number, particle concentration $\phi _p$, and Ca when the following parameters are fixed: (left) $c_r=1.00$, $Ca=1.18\cdot 10^{-3}$, $\varepsilon _n=0.63$, $fr=0.60$; (right) $c_r=1.00$, $\text {Re}=4714$, $\phi _p=6.90\%$, $\varepsilon _n=0.8$, $fr=0.1$.

In addition to blockage dynamics, we evaluated the validity of the model’s response to variation in flow rate (Reynolds number), the concentration of particles, and the granular capillary number^2^ $\text {Ca}={\overline{u}}\mu /\gamma _{ice}$ with ${\overline{u}}$ which is an average flow velocity. These parameters were defined at the inlet of the model. As in the flow map study, to speed up this parametric analysis, we used constant cohesion and restitution coefficients without accounting for lubrication. We aimed to highlight how the blockage time depended on these parameters. The simulation results are presented in Fig. 5. The left plot in this figure illustrates a correlation between the temporal duration of blockage, the Reynolds number, and the concentration of particles. The considered interval of Reynolds numbers is relevant in the food industry (e.g. ice slurries^38^) and represents transient cases in petroleum and mining industries^39^. The findings clearly indicate that when the volume fraction of particles exceeds $4\%$, the blockage time is consistently below 10 s. As the particle concentration rises, it increases the probability of particle interaction and agglomeration. Consequently, this contributes to the rapid formation of blockages within the pipe. The relationship between blockage time and Reynolds number shows the transition point. Prior to reaching a Reynolds number of approximately 6000, a consistent uprising trend is observed. However, beyond this threshold, a reduction in blockage time becomes apparent. Then, at $\text {Re}\approx 8000$, the blockage time dramatically increases, so the blockage takes several hundreds of seconds. The observed trend is rather straightforward as by increasing the Re we increased the relative velocity between the particles and then reduced the efficiency of clogging due to cohesion. However, the number of collisions also increases with Re, which is the reason for the existence of the local minimum blockage time. The right plot in Fig. 5 highlights the significance of the capillary number, which is inversely proportional to adhesion. Reading the plot, it becomes apparent that the blocking time remains mostly within the range of 0–10 s for different capillary number values. The blockage time is inversely proportional to the capillary number. In these simulations we highlighted the influence of cohesion reducing the mechanical deformations ($\varepsilon _n=0.8$) and the friction ($fr=0.1$). We again note a non-linear trend when increasing the cohesion. Namely, at $\text {Ca}\approx 2\cdot 10^{-3}$, the blockage speeds us by a factor of two. This happens due to the enhanced scrubbing of deposited particles from the walls by the clusters of those remaining in the bulk of the flow, promoting partial re-suspension of particles, slugging, and thus bringing more particles to the orifice. When the cohesion increased from this point, the effect is compensated by even more intense deposition.

### Concluding remarks

This study demonstrates that the CFD-DEM approach is capable to reproduce the process of plugging in turbulent multiphase flows with cohesive/adhesive particles with minimal modifications to the standard model. Our model was simplified and based on several assumptions: 2D geometry, low Young modulus, and no influence of the entire experimental system considered. The simulations do however return reasonable results when experimental measurements well support the cohesive properties and the concentration of particles. From the simulations, we found that the inertial collisions and the gravity-driven deposition are the dominant mechanisms leading to plugging the pipe. Although many flow maps are developed to account for these phenomena, the stickiness of the particles, in our case, dislocates plugging towards lower concentrations and higher flow rates on the map. The CFD-DEM method provides excellent insight into the physics of the process. However, due to the high computational costs, which can extend up to 12 h on a system utilizing 30 CPUs of AMD Ryzen Threadripper PRO 3975WX at 3.8 GHz, this approach is hardly applicable for simulating the entire flow system or providing decision support. A more pragmatic yet accurate simulation approach still needs to be developed.

## Data Availability

The datasets used and/or analysed during the current study are available from the corresponding author on reasonable request.
